# Are there gender differences in the trajectories of self-rated health among chinese older adults? an analysis of the Chinese Longitudinal Healthy Longevity Survey (CLHLS)

**DOI:** 10.1186/s12877-021-02484-4

**Published:** 2021-10-18

**Authors:** Shichen Cui, Yushan Yu, Weizhen Dong, Tingke Xu, Yunyun Huang, Xiangyang Zhang, Chun Chen

**Affiliations:** 1 School of Public Health and Management, Wenzhou Medical University, Tongren Building 7B304, Zhejiang 325035 Wenzhou, China; 2 International Centre for Reproductive Health (ICRH), Department of Public Health and Primary Care, Faculty of Medicine and Health Sciences, Ghent University, 9000 Ghent, Belgium; 3 Department of Sociology and Legal Studies, University of Waterloo, 200 University Avenue West, N2L 3G1 Waterloo, Ontario Canada; 4 School of Innovation and Enterpreneurship, Wenzhou Medical University, Zhejiang 325035 Wenzhou, China; 5 The First Affiliated Hospital of Wenzhou Medical University, Zhejiang 325000 Wenzhou, China; 6 Center for Health Assessment, Wenzhou Medical University, 325035 Wenzhou, Zhejiang China

**Keywords:** self-rated health, gender differences, latent growth model, older adults, dropout

## Abstract

**Background:**

Self-rated health (SRH) is a good predictor of morbidity and mortality. Extensive research has shown that females generally report poorer SRH than males but still tend to live longer. Previous studies used cross-sectional or pooled data for their analyses while ignoring the dynamic changes in males’ and females’ SRH statuses over time. Furthermore, longitudinal studies, especially those that focus on older adults, typically suffer from the incompleteness of data. As such, the effect of dropout data on the trajectories of SRH is still unknown. Our objective is to examine whether there are any gender differences in the trajectories of SRH statuses in Chinese older adults.

**Methods:**

The trajectories of SRH were estimated using the pattern-mixture model (PMM), a special latent growth model, under non-ignorable dropout data assumption. We analyzed the Chinese Longitudinal Healthy Longevity Survey (CLHLS) data of 15,613 older adults aged 65 years and above, collected from 2005 to 2014.

**Results:**

The results demonstrated the effect of non-ignorable dropout data assumptions in this study. The previous SRH score was negatively associated with the likelihood of dropping out of the study at the next follow-up survey. Our results showed that both males and females in China perceive their SRH as decreasing over time. A significant gender difference was found in the average SRH score (female SRH was lower than male SRH) in this study. Nonetheless, based on the results obtained using the PMM, there are no gender differences in the trajectories of SRH at baseline as well as in the rate of decline among the total sample. The results also show that males and females respond to SRH predictors similarly, except that current drinking has a more pronounced positive effect on males and healthcare accessibility has a more pronounced positive effect on females.

**Conclusions:**

Our results suggest that missing data have an impact on the trajectory of SRH among Chinese older adults. Under the non-ignorable dropout data assumptions, no gender differences were found in trajectories of SRH among Chinese older adults. Males and females respond to SRH predictors similarly, except for current drinking habit and healthcare accessibility.

**Supplementary Information:**

The online version contains supplementary material available at 10.1186/s12877-021-02484-4.

## Background

The world is rapidly aging. It is predicted that the proportion of people aged 60 and above in the global population will double from 11 % to 2006 to 22 % by 2050[1]. The pace of population aging is much faster in China than in many other countries[2]. According to data published by the National Bureau of Statistics of China, 18.1 % of the total population was aged 60 years and above in 2019[3]. The rapid growth of the elderly population in China has brought about major challenges to public health and social care services. Therefore, simple and valid measures for the evaluation and prediction of health status in older adults are needed to address the financial burden of an aging population on social and health care services[4]. Self-rated health (SRH) has been one of the most frequently used variables in gerontological and health research since the 1950 s[5]. The main advantage of using SRH is that it is one of the most feasible and inclusive measures of peoples’ health statuses[6]. Moreover, it is simple, affordable, and globally implemented. SRH, as a comprehensive health evaluation index, is generally a good predictor of morbidity and mortality among older adults[7]. Considering that females routinely outlive males, it is important to study gender as a basis of differentiation in the study of the elderly population, and specifically, to identify how gender affects SRH[8].

A large body of life-cycle analysis research has shown that females consistently report worse SRH than males, yet still tend to outlive them[9–12]. However, gender differences in SRH among older adults have yet to be properly explored. In general, there are two ways to estimate the SRH between males and females, namely using cross-sectional data or longitudinal data.

Previous studies have used cross-sectional data or pooled data to generalize various patterns of gender differences in SRH. Extensive research has shown no gender differences in SRH of older adults[8, 13–16]. For example, a study of Slovenia, Lithuania, and the UK showed that the most influential factors associated with poor SRH were low education, chronic diseases, inadequate physical activity, and poor mental health, not gender differences[17]. Similarly, the other two studies based on the Chinese Longitudinal Healthy Longevity Survey (CLHLS) suggested no gender differences in SRH[18, 19]. However, some studies using CLHLS data have revealed contradictory findings which show that older males report better SRH than older females[20–24]. Although cross-sectional studies have great explanatory power, they are subject to two limitations. First, cross-sectional studies do not focus on the stability of changes in SRH among older adults. Second, cross-sectional associations tend to overestimate some factors affecting health inequalities[25].

For a decade, an increasing number of longitudinal studies on health inequalities have also been carried out. These studies with two and more time points have shown a slow decline in SRH trajectories across adulthood[25–28]. However, little is known about how gender differences affect the aging process[29]. A few studies have produced mixed results on the direction of the relationship between gender and SRH in older adults. Some suggested no gender difference at all in SRH at baseline, but a faster decline for males in SRH over time[30–33]. Other studies showed that older males tend to report better SRH than older females, but could not distinguish the rate of SRH between them[34]. A Chinese study using CLHLS data supported the latter findings by showing that elderly females reported worse SRH than elderly males, and a decreasing trend of SRH from slow to fast[35]. The existing literature on the trajectory of SRH between older females and males is controversial. Little is known about the factors that influence the trajectory of gender differences in SRH.

Most of the previous studies on the trajectory of gender differences were based on the experience of developed countries, so there is insufficient research on gender differences in developing countries. Furthermore, longitudinal studies, especially those that focused on older adults, typically suffer from the incompleteness of data. Previous longitudinal studies often assumed a missing at random (MAR) mechanism, and they excluded cases with missing data directly for analysis. However, there are many situations in which dropouts are missing not at random (MNAR), especially in research related to older adults, because participants might have died during the study or were too frail to participate in follow-up surveys[36]. When assuming a MAR mechanism, the SRH is associated with mortality among older people, which might lead to some bias in the estimation of the SRH trajectory[37] if the deceased people are included for analysis. However, when assuming an MNAR mechanism, ignoring dropout data can lead to too optimistic inferences, and the growth modeling of longitudinal data with dropout becomes significant[38]. In this case, the effect of dropout data on the trajectories of SRH among Chinese older adults is unknown.

To our knowledge, few studies have examined the longitudinal association between gender and SRH among Chinese older adults. Therefore, this study is crucial in the fight against health inequalities among older adults, which aims to examine gender differences in the trajectories of SRH statuses in Chinese older adults. This study uses data from the CLHLS to examine the following questions: (1) Do the missing data have an impact on the gender trajectory of Chinese older adults? and (2) Do SRH trajectories vary by gender among Chinese older adults?

## Methods

### Data source

**Fig. 1:**
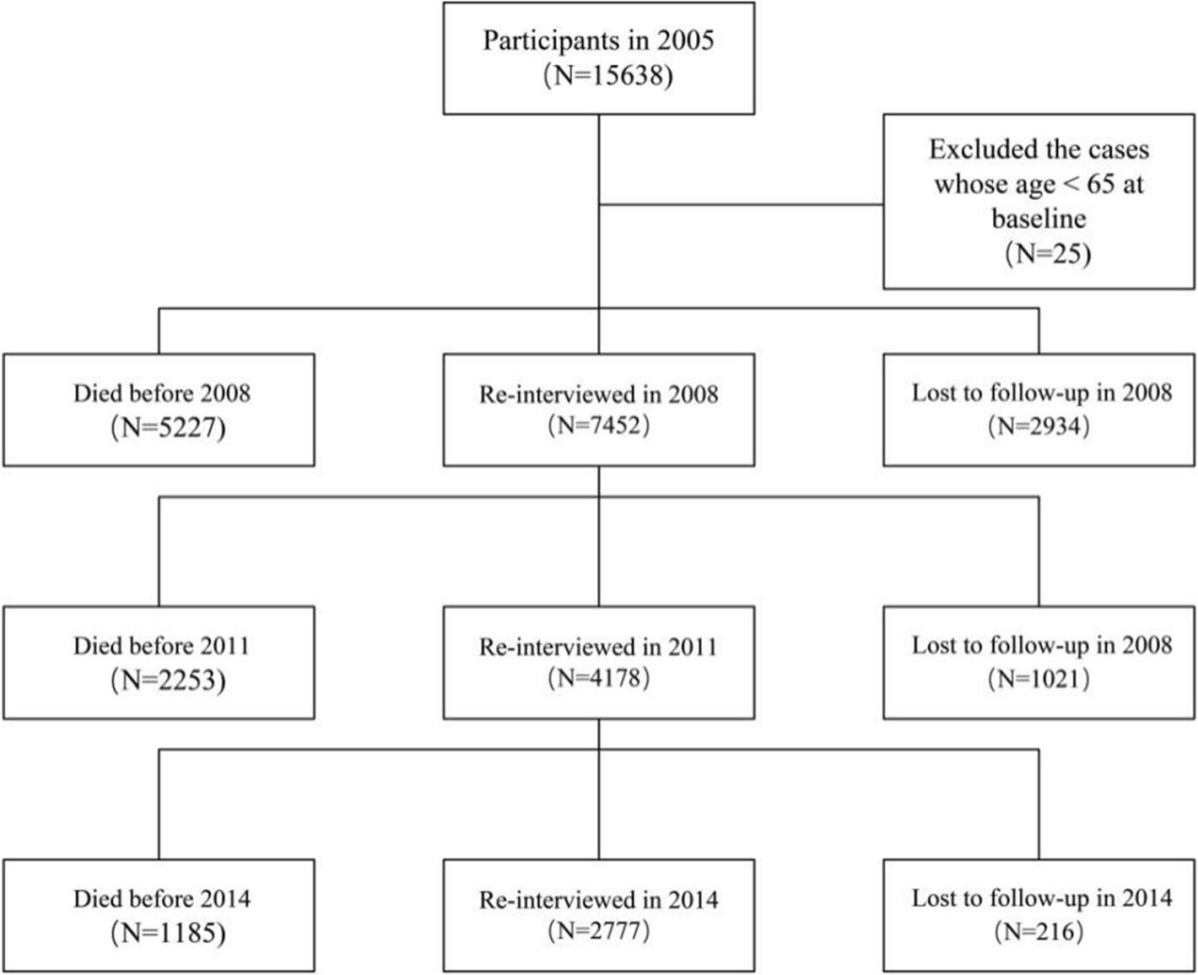
Structure of the study sample

Publicly available data from the CLHLS were used in this study. CLHLS collected information about older adults aged 65 and above regarding their health condition, socioeconomic characteristics, lifestyle, health status, quality of life, mental attitude, daily functioning, health service accessibility, and so on, across 22 provinces in China from 1998 to 2014[39]. The participants were a large, random sample of Chinese older adults. Trained interviewers collected data through face-to-face surveys. The sample design was based on a previous study, and the quality of CLHLS data is good[40]. We treated the 2005 wave data as baseline and excluded newly recruited sample. Finally, 15,163 older adults from 2005 to 2014 were included for analysis after excluding 25 of them aged below 65 years (*N* = 15,613 in 2005, with follow-up interviews completed by 7452 participants in 2008, 4178 in 2011, and 2777 in 2014). Details regarding the study sample are shown in Fig. 1.

### Measurements

#### Dependent variables

SRH is essentially a subjective measure involving complex perceptions of multiple health-related areas[14], which takes into account overall health including physical health conditions, cognitive capacity, psychological well-being, clinical risk factors, health behaviors, etc.[41, 42]. SRH was assessed using the following question: “How do you rate your health at present?” Responses to this question were rated on a five-point Likert scale with the following options: “very good,” “good,” “fair,” “poor,” “very poor,” and “unable to answer.” We recorded “unable to answer” as “missing” in the main analysis. While the reliability of individual ratings was not directly tested in this study, numerous studies have shown that SRH is an accurate measurement of health[5–7, 43–45] and that the meaning of SRH is similar for women and men[15]. Furthermore, our focus on two sensitivity analyses included actions such as dropping cases with “unable to answer” for SRH and recoding the “unable to answer” to “very poor” for SRH. Lower values indicated worse SRH.

#### Independent variables

Based on previous studies[10, 32, 35, 46, 47], the independent variables included time-invariant and time-varying variables. Time-invariant variables included education (primary school or above and illiteracy) and gender (1 = men or 0 = women). Time-varying variables included the socio-structural factors of age, residence (rural or urban), income (ordinal variable), living with a family member (1 = with household members, 0 = without household members), having a spouse (1 = having a spouse, 0 = no spouse), and healthcare accessibility (1 = yes, 0 = no). The behavioral health factors include currently smoking (1 = yes, 0 = no), currently drinking alcohol (1 = yes, 0 = no), participating in social activities (1 = never; 2 = not every month, but sometimes; 3 = not every week, but at least once a month; 4 = not every day, but at least once a week; and 5 = almost every day), the health status factors of being able to perform basic activities of daily living (BADL) (1 = yes, 0 = no), being able to perform instrumental activities of daily living (IADL) (1 = yes, 0 = no), and presence of chronic diseases (measured as the number of chronic diseases). The time-invariant variables were taken from the 2005 wave, with the time-varying ones being taken from the current wave.

### Statistical analysis

**Fig. 2:**
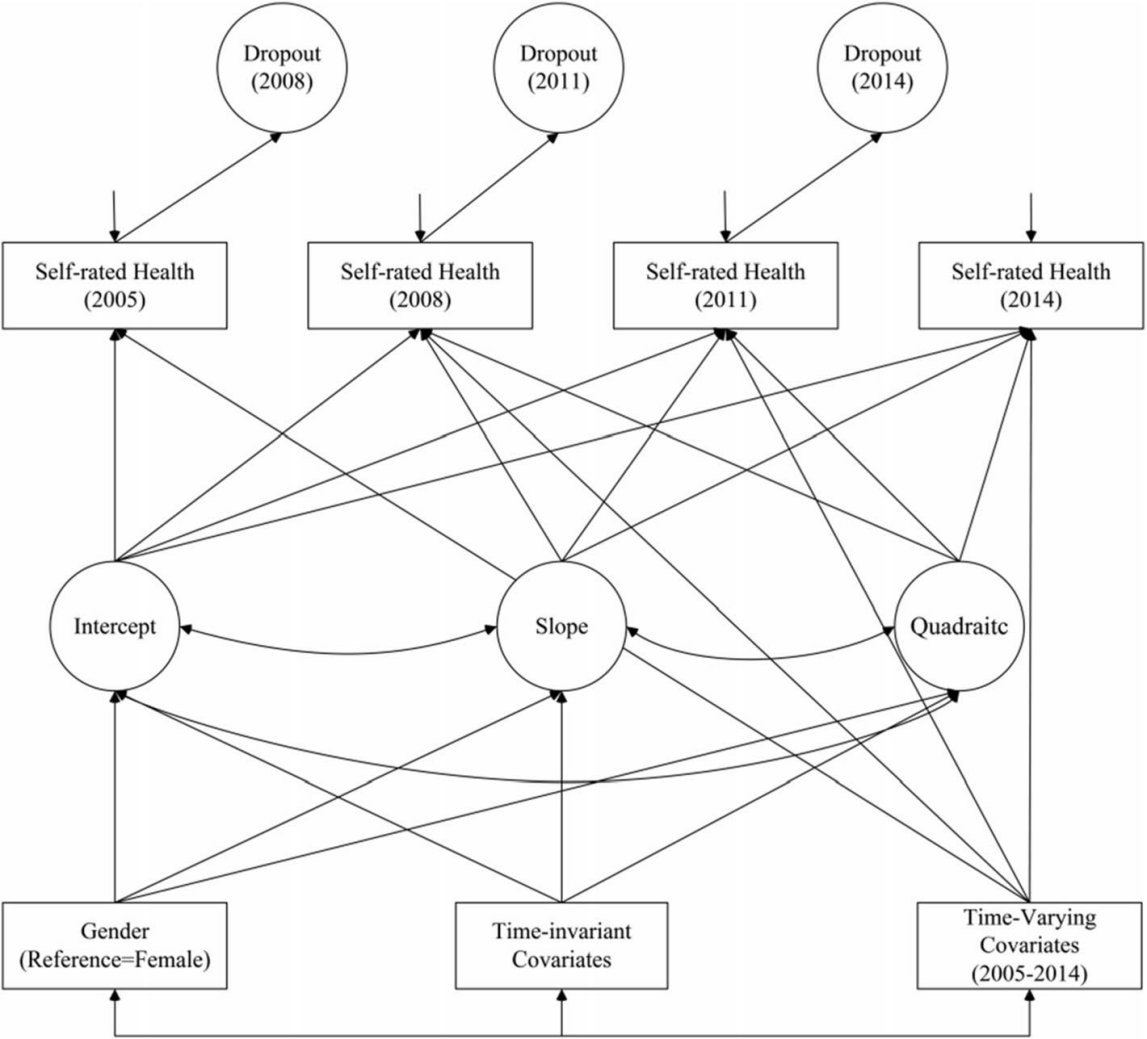
The conceptual latent growth model of this study

Stata 15.1 was used to complete the descriptive statistics. The latent growth model (LGM) analytic approach (Fig. 2), which was performed in Mplus 8.4, was used to estimate the SRH trajectories. LGM is effective at analyzing repeated measures of longitudinal data and can predict the growth trajectory of the outcome via latent intercepts and slopes[48]. In this model, the intercept growth factor is referred to as the initial status when the time score is zero, with the slope growth factor referring to the growth rate of the time score increase of one unit[49]. In this study, persons with rapidly declining SRH may be more likely to die or become too frail to participate. Thus, the missingness for those who dropped out is not random in this because the drop out is related to both past and current outcome, and this missingness is considered non-ignorable. The results from directly using LGM will be misleading unless this non-ignorable missingness is addressed in the model[50]. Pattern-mixture models (PMM) have been used in many applications when longitudinal non-ignorable missingness is a concern[51, 52]. Thus, a PMM was used to estimate growth models for the outcome with dropout indicators in our study. The study included both the time-invariant and time-varying variables in this model.

**Fig. 3:**
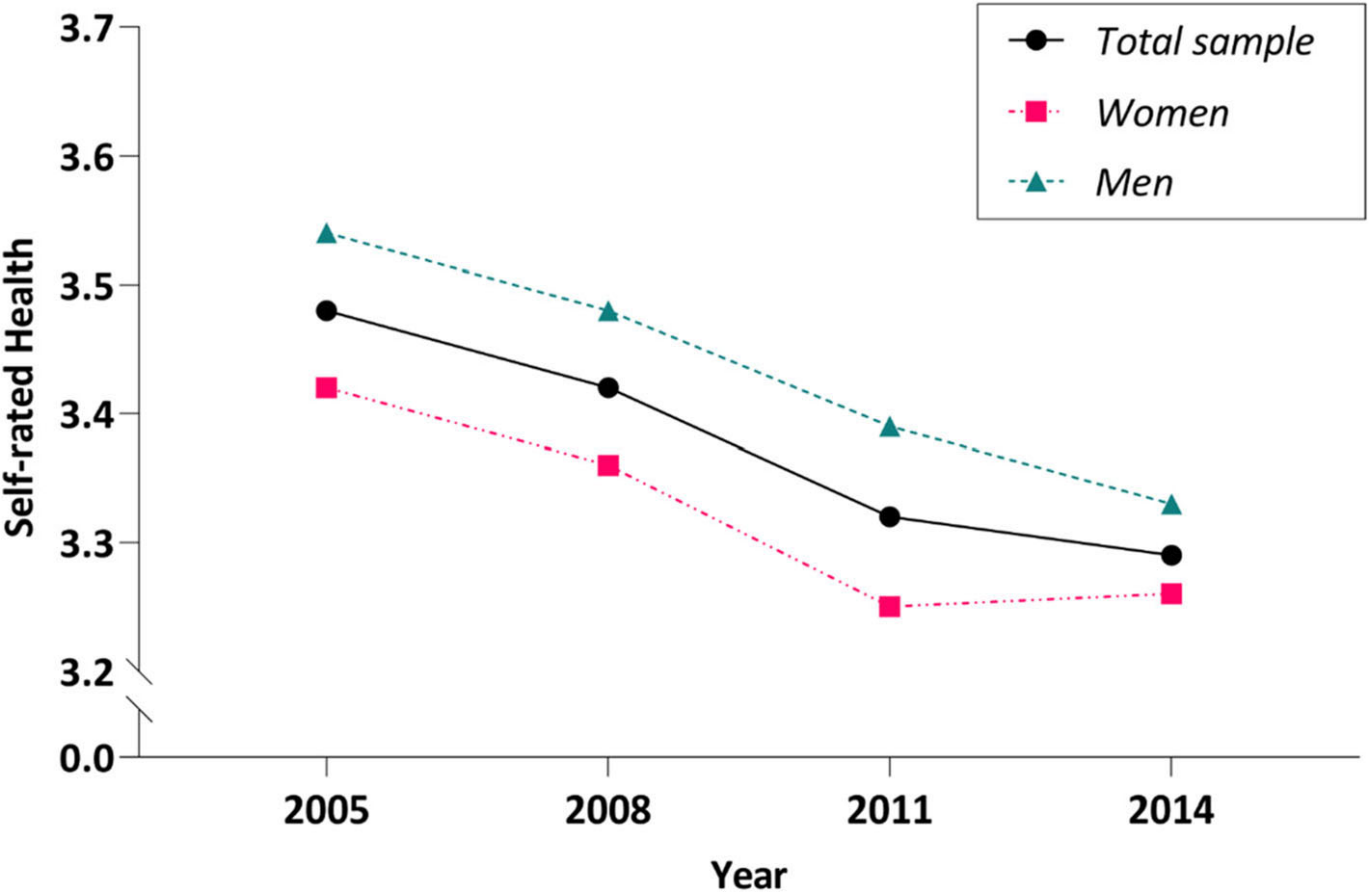
SRH score changes over time

We undertook four steps to examine our assumption, as follows: (1) Descriptive statistics of SRH were computed, including mean and standard variance. (2) The mean plots for the sample and estimated means were calculated to determine the shape of the growth curve (Fig. 3). A linear LGM (Model A), a free time scores LGM (Model B), and a quadratic LGM (Model C) were utilized to examine the growth trajectory of SRH. (3) Based on selected best fit model, gender, covariates and dropout indicators were added, the pattern-mixture model was built. (4) To examine the gender difference, gender subgroup (male and female) PMMs were employed.

The following three sensitivity analysis methods were used to test the robustness of the results. We considered “unable to answer” as being designed for participants who were too frail to answer the self-rated question in CLHLS[53]. First, we performed sensitivity analysis after dropping cases with “unable to answer” for SRH. Then, we performed sensitivity analysis again after recoding the “unable to answer” to “very poor” for SRH. After applying the non-ignorable missing data mechanism in our main results, we found our missing data included “lost to follow-up” and “dead” samples, so we performed sensitivity analysis again after dropping the “lost to follow-up” cases.

For handling the missing data, two methods were introduced: (1) replacing missing values of independent variables with non-missing values of the previous year and (2) using maximum likelihood estimation under MNAR to reduce the potential bias from missing data.

## Results

### Descriptive statistics of the sample

In total, 15,613 older adults [female: 8934 (57.2 %), male: 6679 (42.8 %)] aged 65 to 120 years old were included in our study at the baseline. Given that the CLHLS was specially designed by considering the clusters of age-sex residence (urban/rural), the weight variable was applied to calculate the descriptive statistics for the whole elderly population in the sampled provinces[54]. After controlling for weightages, there were 16,093 older adults in total, 8382 (52.1 %) of whom were female and 7711 (47.9 %) were male.

A significant gender difference was found in the average SRH of all four waves of data  (Fig. 3). The mean scores of SRH of males were 3.54 ± 0.93, 3.48 ± 0.96, 3.39 ± 0.95, and 3.33 ± 0.93 at years 2005, 2008, 2011, and 2014, respectively, which were significantly higher than females’ 3.42 ± 0.93, 3.36 ± 0.94, 3.25 ± 1.00, and 3.26 ± 0.92 at years 2005, 2008, 2011, and 2014, respectively (2005: *P* < 0.001; 2008: *P* < 0.001; 2011: *P* < 0.001; 2014: *P* = 0.005).

In addition, we found a gender difference in the socio-structural factors, behavioral health factors, and health status factors except for healthcare accessibility and residence. A description of the full sample and subgroups can be found in Table 1.

**Table 1:** Descriptive statistics of full sample and subgroups

		N (%) / Mean ± SD / Median (Interquartile Range) (*N*=16093)	Female (*N* = 8382)	Male (*N* = 7711)	*P-value*
***Social structural factors at baseline***					
Age at baseline***		72.52 ± 6.01	72.97 ± 6.28	72.04 ± 5.66	< 0.001
Education***					< 0.001
	Illiteracy	7390 (45.99)	5515 (65.93)	1875 (24.34)	
	Primary school or above	8678 (54.01)	2850 (34.07)	5828 (75.66)	
	Missing	25	17	8	
Residence^ns^					0.355
	Rural	9210 (57.23)	4768 (56.88)	4442 (57.61)	
	Urban	6883 (42.77)	3614 (43.12)	3269 (42.39)	
	Missing	0	0	0	
Income***		2.99 ± 0.65	2.96 ± 0.64	3.01 ± 0.67	< 0.001
Living with family member***					< 0.001
	No	2238 (13.91)	1371 (16.36)	867 (11.25)	
	Yes	13,851 (86.09)	7009 (83.64)	6842 (88.75)	
	Missing	4	2	2	
Have a spouse***					< 0.001
	No	6129 (38.09)	4299 (51.29)	1830 (23.73)	
	Yes	9963 (61.91)	4082 (48.71)	5881 (76.27)	
	Missing	1	1	0	
Healthcare accessibility^ns^					0.217
	No	1500 (9.32)	804 (9.59)	696 (9.03)	
	Yes	14,593 (90.68)	7578 (90.41)	7015 (90.97)	
	Missing	0	0	0	
***Behavioral health factors at baseline***					
Current smoking***					< 0.001
	No	11,737 (72.96)	7596 (90.66)	4141 (53.72)	
	Yes	4350 (27.04)	783 (9.34)	3567 (46.28)	
	Missing	6	3	3	
Current drinking***					< 0.001
	No	12,276 (76.30)	7541 (89.97)	4735 (61.43)	
	Yes	3814 (23.70)	841 (10.03)	2973 (38.57)	
	Missing	3	0	3	
Social activities ***		1.48 ± 1.06	1.40 ± 0.97	1.58 ± 1.13	< 0.001
***Health status factors at baseline***					
BADL**					0.006
	No	15,094 (93.86)	7819 (93.36)	7275 (94.39)	
	Yes	988 (6.14)	556 (6.64)	432 (5.61)	
	Missing	11	7	4	
IADL***					< 0.001
	No	10,334 (64.26)	4766 (56.87)	5568 (72.29)	
	Yes	5748 (35.74)	3614 (43.13)	2134 (27.71)	
	Missing	11	2	9	
Chronic diseases***		1.00 (0.00 ~ 2.00)	1.00 (0.00 ~ 2.00)	1.00 (0.00 ~ 1.00)	< 0.001
***Self-rated Health***					
	2005***	3.48 ± 0.93	3.42 ± 0.93	3.54 ± 0.93	< 0.001
	2008***	3.42 ± 0.95	3.36 ± 0.94	3.48 ± 0.96	< 0.001
	2011***	3.32 ± 0.98	3.25 ± 1.00	3.39 ± 0.95	< 0.001
	2014**	3.29 ± 0.93	3.26 ± 0.92	3.33 ± 0.93	0.005

Note: *p < 0.05. **p < 0.01. ***p < 0.001; ns(no significant)

### Model selection

We performed model selection based on the change trend of the four-wave SRH mean value of the full sample ( Fig. 3). We examined the indicators of model fit for these unconditional growth models (Table 2), which included the linear growth model (Model A), the free time score growth model (Model B), and the quadratic growth model (Model C). The results indicated that Model C had a higher CFI and TLI than the other two models, while Model C had a lower RMSEA, SRMR, and χ^2^/df. As a result, the quadratic growth model best fits our data. Taking into account that poor SRH could lead to the dropout of each participant, the dropout indicator was added to the quadratic growth model to construct the PMM.

**Table 2:** Model fit information and comparisons

*Model*	*Number of parameters*	*Chi-Square Test of Model Fit*	*df*	*P-value*	*AIC*	*BIC*	*RMSEA (95% CI)*	*CFI*	*TLI*	*SRMR*
**Model A**	9	16.481	5	0.0056	73979.671	74047.909	0.013 (0.006 ~ 0.020)	0.986	0.983	0.021
**Model B**	11	11.503	3	0.0093	73978.114	74061.516	0.014 (0.006 ~ 0.023)	0.990	0.979	0.014
**Model C**	13	0.127	1	0.7213	73970.919	74069.484	<0.001 (0.000 ~ 0.016)	1.000	1.006	0.002

Note: Model A was the unconditional linear LGM; Model B was the unconditional linear LGM with free time scores; Model C was the unconditional quadratic LGM

### Results of pattern-mixture model

Table 3 presents the main results obtained using the PMM. The full results of PMM can view the appendix Table 1. The results of SRH on dropout status were consistent with our model assumption that SRH is negatively associated with the dropout. For example, given that the SRH at 2005 was negatively associated with dropout at 2008[*β *= -0.054 (95 %CI:-0.082~-0.027), *P* < 0.001], the lower SRH at 2008 could be at a high risk of dropout at 2011 [*β *= -0.130 (95 %CI:-0.171~-0.089)], *P* < 0.001], except for the fact that the SRH at 2011 was not associated with the dropout at 2014[*β *= -0.016 (95 %CI:--0.093 ~ 0.061), *P* = 0.688].

For gender, the difference of SRH at baseline between male and female was not significant [*β* = 0.033 (95 %CI: -0.055 ~ 0.121), *P* = 0.461]. In additional, gender (male vs. female) could not predict the slope [*β *=-0.028 (95 %CI: -0.251 ~ 0.194), *P* = 0.803] or quadratic [*β *= -0.039 (95 %CI: -0.191 ~ 0.159), *P* = 0.610] growth of SRH trajectories.

For socio-structural factors, education could not predict the intercept, slope, or quadratic growth. However, age, income, and residence (rural vs. urban) were positively associated with SRH in four study periods expect the residence in 2011. As to lifestyle, smoking was positively associated with SRH in 2005, while drinking was positively associated with SRH in 2005, 2011 and 2014. We also noted that higher level of social activities may result in better SRH among the four study periods. For social relationship, living arrangement (living with family members vs. living alone) was not associated with SRH in the four study periods, while marital status was only associated with SRH at 2005. For health status, ADL disability, IADL disability, and chronic diseases were negatively associated with SRH. For health services accessibility, adequate healthcare accessibility was positively associated with a better SRH.

**Table 3:** Main results of the pattern-mixture model

*Variables*		Total Sample	Male	Female
		*β* **(95 %CI)**	*β* **(95 %CI)**	*β* **(95 %CI)**
***Time-invariant variables***				
	Male	0.033 (-0.055 ~ 0.121)		
	Primary school or above	-0.043 (-0.127 ~ 0.04)	0.004 (-0.037 ~ 0.044)	-0.054 (-0.128 ~ 0.019)
***Slope***				
	Male	-0.028 (-0.251 ~ 0.194)		
	Primary school or above	0.017 (-0.183 ~ 0.217)	-0.016 (-0.075 ~ 0.043)	0.035 (-0.092 ~ 0.162)
***Quadratic***				
	Male	-0.039 (-0.191 ~ 0.112)		
	Primary school or above	0.021 (-0.116 ~ 0.159)	0.018 (-0.053 ~ 0.088)	0.005 (-0.119 ~ 0.128)
***Time-varying variables***				
***Self-rated health →dropout***				
	SRH2005→ droput2008	-0.054 (-0.082~-0.027) ***	-0.065 (-0.107~-0.023) **	-0.043 (-0.080~-0.006) *
	SRH2008 → droput2011	-0.130 (-0.171~-0.089) ***	-0.194 (-0.255~-0.134) ***	-0.078 (-0.133~-0.022) **
	SRH2011 → droput2014	-0.016 (-0.093 ~ 0.061)	-0.058 (-0.172 ~ 0.056)	0.019 (-0.088 ~ 0.126)

Note: β and 95 % CI in the table above. *p < 0.05, **p < 0.01, ***p < 0.001

### Results of subgroups pattern-mixture model

Table 3 presents results of gender subgroups pattern-mixture model. To examine the gender difference, analysis was also conducted separately to compare males and females to fit the subgroups pattern-mixture model. We observed a similarity in both female and male subgroups analysis: first, as to socio-structural factors, education could not predict the intercept, slope, or quadratic growth of SRH; the residence (rural vs. urban) was positively associated with SRH in 2008; age and income were positively associated with SRH. Second, as to lifestyle, higher level of social activities may result in a better SRH among the four study periods. Third, as to health status, ADL disability, IADL disability and chronic diseases were negatively associated with SRH.

We found the gender difference as follows: First, drinking was positively associated with SRH, except for the year 2008 in the male subgroup, while drinking was positively associated with SRH only at 2008 in the female subgroup. Second, as to health services accessibility, adequate healthcare accessibility was positively associated with a better SRH in the four study periods in the female subgroup, and was only significant in 2008 and 2014 in the male subgroup.

### Results of sensitivity analysis

We conducted sensitivity analysis in three ways: (1) eliminating cases with “unable to answer” for SRH, (2) recoding the “unable to answer” to “very poor” for SRH, and (3) eliminating the lost to follow-up cases. As in our main results, gender could not predict the intercept, slope, or quadratic growth of SRH. The three sensitivity analyses showed the same results (Table 4), which are consistent with our main results.

**Table 4:** Sensitivity analysis of this study

	*Sensitivity analysis A*		*Sensitivity analysis B*		*Sensitivity analysis C*	
	***β (95 %CI)***	***P-value***	***β (95 %CI)***	***P-value***	***β (95 %CI)***	***P-value***
Intercept of gender	0.033 (-0.064 ~ 0.129)	0.507	0.026 (-0.057 ~ 0.109)	0.538	0.093 (-0.054 ~ 0.239)	0.214
Slope of gender	-0.088 (-0.571 ~ 0.395)	0.721	-0.038 (-0.262 ~ 0.187)	0.742	-0.001 (-0.326 ~ 0.325)	0.997
Quadratic of gender	-0.021 (-0.196 ~ 0.154)	0.818	-0.006 (-0.131 ~ 0.12)	0.928	-0.045 (-0.198 ~ 0.109)	0.569

Note: Sensitivity analysis A: dropping cases with “unable to answer” for SRH; Sensitivity analysis B: recoding the “unable to answer” to “very poor” for SRH; Sensitivity analysis C: dropping the cases who lost-to-follow up.

## Discussion

This study used a quadratic PMM to examine two questions: (1) Do the missing data have an impact on the gender trajectory of the Chinese older adults? and (2) Do SRH trajectories vary by gender among Chinese older adults? The data were from a national random sample of older adults aged 65 years and above across 22 provinces in China, collected from 2005 to 2014. Four main results can be discussed.

First, this study supports the argument that gender differences in SRH trajectory among older adults needs to consider the impact of dropout data. Previous longitudinal studies based on older adults often did not explicitly illustrate the missing data[16, 30, 31, 35], or deleted the cases with missing data directly, which often assume a missing at random (MAR) mechanism. Due to the longitudinal nature of the data, there is the problem of sample loss, which may introduce serious deviations in any analysis. This study showed that previous SRH score was negatively associated with the likelihood of dropping out of the study at the next follow-up survey. Generally speaking, longitudinal studies focusing on older adults often suffered from the incompleteness of data since subjects who initially rejoined the study failed to participate in one or more subsequent waves. In this study, dropout was missing not at random because dropout was related to past outcome. Previous studies suggested that including decreased people would lead to sampling errors and bias in the estimation of the outcome trajectory, especially if SRH is associated with mortality[37]. So, PMM are important options to consider, especially when outcome-related dropout seems believable[36].

Second, under non-ignorable dropout data assumptions, no gender differences were found in trajectories of SRH among Chinese older adults. Nonetheless, the role of gender in SRH related longitudinal analysis research remains ambiguous[30, 55]. For example, an American study using data that spanned 12 years (1992–2004) found among middle and old age adults that there was no gender difference in SRH at baseline and that SRH declined faster for males over time[32]. Similarly, a Chinese longitudinal study using data that spanned 12 years (2002–2014) found that elderly females had a worse initial state of SRH than did male elders[35]. Unlike prior studies on SRH trajectories, this study showed that both males and females in China perceive their health as decreasing over time, but there was no gender difference in SRH at baseline and in the rate of decline among the total sample. Conflicting results may be due to sampling population difference or researching time differences in those studies, or methodological problems such as ignoring missing data.

Third, although there is no gender difference in the trajectories, the average SRH of females is lower than that of males. The present study showed that higher income, greater participation in social activities, age, current drinking, residence (rural vs. urban), and healthcare accessibility are positively associated with the SRH of older adults, while poor health status was negatively associated with both males and female SRH across almost every survey time point. Thus, social disadvantage might be an important reason for females’ poorer SRH. For example, a high SRH score is associated with a higher socioeconomic status, i.e., generally male tender to with more social resources, so they are more likely to be in good health status in our society[46, 56]. Gender differences also exist in the exposure to physical activity, cognitive capacity, various lifestyle behaviors, and psychosocial factors[46, 57]. Good follow-up of SRH is also related to having greater social support[58], with poor follow-up of SRH being predicted by one’s health status (e.g., having a high number of illnesses, low levels of physical activity, difficulties in activities of daily living)[31, 59].

Finally, performing the analysis by gender of the respondent, the results show that males and females respond to SRH predictors similarly. Education could not predict the intercept, slope, or quadratic of SRH; age, income, health services, and social activities are positively associated with both males and female SRH, while health status factors were negatively associated with both male and female SRH. The result is consistent with most studies on SRH trajectories in the general adult population or aging population[15, 25, 29, 46]. However, our study found current drinking to have a more pronounced positive effect on males and health services to have a more pronounced positive effect on females. Recent research showed that alcohol consumption can’t bring significant health benefits[60]. The reason of current drinking differentially affected these two genders might be males, in the higher level of health status, are more prefer to drink alcohol comparing with females in China[61]. In this study, although there is no difference in healthcare accessibility between males and females, the positive effect of health services accessibility on females’ SRH assessment is more obvious. It might be because when in the same health services accessibility, females have higher medical care service utilization[62], which might benefit to their health maintenance.

## Study limitations and recommendations for future research

There were several limitations to this research. First, this study used only SRH measurement. Health indicators are multidimensional, including both objective and subjective factors. Hence, future studies should include objective measures to assess health based on gender. Second, psychosocial factors are also patterned according to gender. Peoples’ experiences of chronic stress and their level of psychological resources are rooted in the socio-structural context of their lives. Future studies should introduce psychological variables to test gender differences in health statuses. Third, this study was unable to eliminate the possibility of reverse causality. Although this study lagged variables when possible, it is possible that the SRH affected the covariates included in the analysis. For example, because changes in current drinking are taking place at the same time as changes in SRH, it is possible that changes in SRH influenced current drinking. Finally, interdisciplinary research needs to be strengthened, as health studies have failed to adequately explore the combination of social and biological aspects in the differences between male and female health statuses.

## Conclusions

The results of this study showed that (1) missing data had an impact on the gender trajectory of the Chinese older adults, and SRH was negative associated with the dropout (2) under non-ignorable dropout data assumptions, no gender differences were found in SRH trajectories among Chinese older adults (3) males and females respond to SRH predictors similarly, except for current drinking and health services. Based on these results, we make the following recommendations for future policymaking and service implementation efforts. First, the health level of older adults in China has shown a declining trend, so the government and the local care communities should provide more health services to meet their health needs. Second, public policies must continue to address the cause and consequences of the social disadvantages that older females face. Third, public policy makers should pay attention to the health needs of both males and females, who may be at an increased risk of disability and illness. Finally, the management of health status should be strengthened, the accessibility of health services should be improved, and gender inequality should be reduced since these factors are conducive to improving the health of both genders.

## Supplementary information

Additional file 1: Appendix Table 1

## Data Availability

All data used in this study were stored at http://opendata.pku.edu.cn and available upon request. If someone wants to request the data from this study, please contact opendata@lib.pku.edu.cn.

## Abbreviations

SRH: Self-Rated Health
LGM: latent growth model
CLHLS: Chinese Longitudinal Healthy Longevity Survey
BADL: basic activities of daily living
IADL: instrumental activities of daily living
PPM: pattern-mixture model
