# Supplementary material for: Are there gender differences in the trajectories of self-rated health among chinese older adults? an analysis of the Chinese Longitudinal Healthy Longevity Survey (CLHLS)
Source: BMC Geriatr. 2021 Oct 18;21:563. doi: 10.1186/s12877-021-02484-4 (PMC8522225; doi:10.1186/s12877-021-02484-4)
Supplement: Supplementary file 1 — Appendix Table 1 [file 12877_2021_2484_MOESM1_ESM.docx]

***Appendix Table1 Results of the pattern-mixture model (Full Model)***

| ***Variables*** | | **Total Sample** | **Male** | **Fmale** |
| --- | --- | --- | --- | --- |
|  |  | **β(95%CI)** | **β(95%CI)** | **β(95%CI)** |
| ***Time-invariant variables*** | |  |  |  |
|  | |  |  |  |
|  | Male | 0.033(-0.055~0.121) |  |  |
|  | Primary school or above | -0.043(-0.127~0.04) | 0.004(-0.037~0.044) | -0.054(-0.128~0.019) |
| ***Slope*** | |  |  |  |
|  | Male | -0.028(-0.251~0.194) |  |  |
|  | Primary school or above | 0.017(-0.183~0.217) | -0.016(-0.075~0.043) | 0.035(-0.092~0.162) |
| ***Quadratic*** | |  |  |  |
|  | Male | -0.039(-0.191~0.112) |  |  |
|  | Primary school or above | 0.021(-0.116~0.159) | 0.018(-0.053~0.088) | 0.005(-0.119~0.128) |
| ***Time-varying variables*** | |  |  |  |
| ***Social structural factors*** | |  |  |  |
|  | *Age 2005* | 0.078(0.048~0.109) *** | 0.099(0.056~0.142) *** | 0.056(0.013~0.100) * |
|  | Age 2008 | 0.068(0.029~0.108) *** | 0.056(0.000~0.111) * | 0.078(0.023~0.133) ** |
|  | Age 2011 | 0.089(0.047~0.132) *** | 0.092(0.031~0.154) ** | 0.079(0.022~0.136) ** |
|  | Age 2014 | 0.128(0.069~0.186) *** | 0.145(0.063~0.227) *** | 0.111(0.030~0.193) ** |
|  | Urban 2005 | 0.024(0.001~0.047) * | 0.024(-0.010~0.058) | 0.025(-0.006~0.057) |
|  | Urban 2008 | 0.063(0.034~0.092) *** | 0.052(0.009~0.095) * | 0.075(0.035~0.115) *** |
|  | Urban 2011 | 0.014(-0.018~0.047) | 0.002(-0.046~0.049) | 0.026(-0.019~0.071) |
|  | Urban 2014 | 0.040(0.005~0.074) * | 0.048(-0.002~0.098) | 0.037(-0.012~0.085) |
|  | Income 2005 | 0.193(0.168~0.218) *** | 0.194(0.157~0.231) *** | 0.188(0.154~0.222) *** |
|  | Income 2008 | 0.224(0.192~0.255) *** | 0.234(0.188~0.281) *** | 0.212(0.169~0.255) *** |
|  | Income 2011 | 0.235(0.202~0.268) *** | 0.214(0.166~0.262) *** | 0.258(0.206~0.295) *** |
|  | Income 2014 | 0.210(0.172~0.248) *** | 0.212(0.160~0.265) *** | 0.205(0.151~0.268) *** |
|  | Living with family members 2005 | 0.008(-0.015~0.032) | -0.010(-0.045~0.025) | 0.021(-0.011~0.053) |
|  | Living with family members 2008 | 0.001(-0.031~0.032) | -0.020(-0.069~0.028) | 0.009(-0.032~0.051) |
|  | Living with family members 2011 | -0.012(-0.048~0.024) | 0.009(-0.045~0.063) | -0.019(-0.067~0.029) |
|  | Living with family members 2014 | 0.014(-0.021~0.049) | 0.032(-0.024~0.088) | 0.002(-0.044~0.047) |
|  | have a spouse 2005 | -0.043(-0.071~-0.015) ** | -0.008(-0.048~0.032) | -0.066(-0.102~-0.031) *** |
|  | have a spouse 2008 | -0.008(-0.043~0.027) | 0.013(-0.040~0.066) | -0.005(-0.048~0.038) |
|  | have a spouse 2011 | -0.010(-0.048~0.028) | -0.024(-0.083~0.035) | -0.016(-0.062~0.029) |
|  | have a spouse 2014 | -0.019(-0.059~0.021) | 0.007(-0.050~0.065) | -0.04(-0.091~0.010) |
|  | healthcare accessibility 2005 | 0.091(0.066~0.116) *** | 0.075(0.037~0.114) *** | 0.104(0.071~0.137) *** |
|  | healthcare accessibility 2008 | 0.042(0.010~0.073) ** | 0.002(-0.042~0.046) | 0.071(0.027~0.115) ** |
|  | healthcare accessibility 2011 | 0.058(0.016~0.100) ** | 0.051(-0.002~0.103) | 0.069(0.004~0.134) * |
|  | healthcare accessibility 2014 | 0.094(0.050~0.139) *** | 0.066(0.005~0.127) * | 0.109(0.045~0.173) *** |
| ***Behavioral health factors*** | |  |  |  |
|  | current smoking 2005 | 0.028(0.004~0.052) * | 0.017(-0.017~0.050) | 0.035(0.005~0.065) * |
|  | current smoking 2008 | 0.015(-0.015~0.045) | 0.024(-0.018~0.065) | 0.018(-0.025~0.061) |
|  | current smoking 2011 | 0.019(-0.014~0.052) | -0.010(-0.056~0.036) | 0.032(-0.019~0.083) |
|  | current smoking 2014 | -0.006(-0.043~0.031) | 0.002(-0.050~0.053) | -0.024(-0.078~0.029) |
|  | current drinking 2005 | 0.049(0.026~0.071) *** | 0.053(0.020~0.086) ** | 0.036(0.008~0.065) * |
|  | current drinking 2008 | 0.020(-0.010~0.050) | 0.022(-0.020~0.063) | 0.027(-0.014~0.069) |
|  | current drinking 2011 | 0.060(0.027~0.092) *** | 0.079(0.031~0.126) *** | 0.024(-0.020~0.067) |
|  | current drinking 2014 | 0.039(0.002~0.077) * | 0.062(0.011~0.113) * | 0.01(-0.049~0.068) |
|  | social activities 2005 | 0.063(0.041~0.085) *** | 0.044(0.013~0.076) ** | 0.079(0.048~0.110) *** |
|  | social activities 2008 | 0.085(0.054~0.117) *** | 0.108(0.064~0.152) *** | 0.064(0.019~0.109) ** |
|  | social activities 2011 | 0.075(0.043~0.108) *** | 0.071(0.022~0.120) ** | 0.08(0.035~0.125) *** |
|  | social activities 2014 | 0.073(0.039~0.108) *** | 0.057(0.004~0.111) * | 0.091(0.046~0.136) *** |
| ***Health status factors*** | |  |  |  |
|  | BADL disability 2005 | -0.104(-0.132~-0.076) *** | -0.098(-0.139~-0.057) *** | -0.107(-0.145~-0.069) *** |
|  | BADL disability 2008 | -0.099(-0.144~-0.053) *** | -0.088(-0.153~-0.023) ** | -0.106(-0.167~-0.044) *** |
|  | BADL disability 2011 | -0.065(-0.104~-0.025) *** | -0.068(-0.126~-0.009) * | -0.062(-0.115~-0.009) * |
|  | BADL disability 2014 | -0.053(-0.093~-0.013) ** | -0.083(-0.140~-0.026) ** | -0.024(-0.080~0.032) |
|  | IADL disability 2005 | -0.183(-0.210~-0.156) *** | -0.202(-0.243~-0.161) *** | -0.159(-0.194~-0.123) *** |
|  | IADL disability 2008 | -0.190(-0.225~-0.156) *** | -0.190(-0.244~-0.136) *** | -0.182(-0.225~-0.138) *** |
|  | IADL disability 2011 | -0.176(-0.212~-0.139) *** | -0.175(-0.230~-0.120) *** | -0.158(-0.205~-0.110) *** |
|  | IADL disability 2014 | -0.214(-0.252~-0.175) *** | -0.214(-0.272~-0.155) *** | -0.205(-0.255~-0.156) *** |
|  | chronic diseases 2005 | -0.201(-0.226~-0.176) *** | -0.185(-0.222~-0.147) *** | -0.213(-0.246~-0.180) *** |
|  | chronic diseases 2008 | -0.211(-0.243~-0.179) *** | -0.215(-0.266~-0.163) *** | -0.205(-0.246~-0.164) *** |
|  | chronic diseases 2011 | -0.189(-0.222~-0.155) *** | -0.194(-0.247~-0.142) *** | -0.201(-0.243~-0.158) *** |
|  | chronic diseases 2014 | -0.229(-0.264~-0.193) *** | -0.240(-0.291~-0.189) *** | -0.217(-0.266~-0.167) *** |
| ***Self-rated health →dropout*** | |  |  |  |
|  | SRH2005→ droput2008 | -0.054(-0.082~-0.027) *** | -0.065(-0.107~-0.023) ** | -0.043(-0.080~-0.006) * |
|  | SRH2008 → droput2011 | -0.130(-0.171~-0.089) *** | -0.194(-0.255~-0.134) *** | -0.078(-0.133~-0.022) ** |
|  | SRH2011 → droput2014 | -0.016(-0.093~0.061) | -0.058(-0.172~0.056) | 0.019(-0.088~0.126) |
| ***Correlation*** | |  |  |  |
|  | Slope with intercept | -0.136(-1.717~1.445) | -0.830(-0.888~-0.773) *** | -0.673(-1.025~-0.321) *** |
|  | Quadratic with intercept | 0.061(-0.810~0.933) | 0.736(0.648~0.824) *** | 0.495(0.111~0.888) * |
|  | Quadratic with slope | -0.902(-1.123~-0.681) *** | -0.973(-0.988~-0.958) *** | -0.901(-0.972~-0.830) *** |

Note: β and 95% CI in the table above. *p <0.05, **p <0.01, ***p <0.001.
